# Active Function Improvement with Repeated Injections of Botulinum Toxin A in Upper Limb Spasticity: A Longitudinal Analysis from the ULIS-III Study

**DOI:** 10.3390/toxins18100429

**Published:** 2026-10-02

**Authors:** Stephen A. Ashford, Mark Weatherall, Klemens Fheodoroff, Jorge Jacinto, Richard J. Siegert, Christian Hannes, Lynne Turner-Stokes

**Affiliations:** 1 Department of Palliative Care, Policy and Rehabilitation, Cicely Saunders Institute, Florence Nightingale Faculty of Nursing, Midwifery and Palliative Care, King’s College London, London SE5 9PJ, UK; lynne.turner-stokes@nhs.net; 2 Regional Hyper-Acute Rehabilitation Unit, Northwick Park Hospital, London North West University Hospitals, London HA1 3UJ, UK; 3 Faculty of Medicine, University of Otago, Dunedin 9016, New Zealand; mark.weatherall@otago.ac.nz; 4 Neurorehabilitation, Gailtal-Klinik, 9620 Hermagor, Austria; klemens.fheodoroff@me.com; 5 Centro de Medicina de Reabilitaçãode Alcoitão, Serviço de Reabilitação de Adultos 3, 2649-506 Estoril, Portugal; jor.jacinto@netcabo.pt; 6 Department of Psychology and Neuroscience, Faculty of Health and Environmental Science, Auckland University of Technology, Auckland 0627, New Zealand; richard.siegert@aut.ac.nz; 7 Ipsen, Einsteinstraße 174, 81677 Munich, Germany; christian.hannes@ipsen.com

**Keywords:** active function, botulinum toxin, upper limb spasticity, goal attainment scaling, rehabilitation

## Abstract

Improvement in active function (making it easier to use the limb) is a common goal following treatment of upper limb spasticity with botulinum toxin (BoNT-A). ULIS-III was a large international, observational, longitudinal study (N = 953) conducted in the context of usual clinical practice over two years. In this secondary post hoc analysis, we examine whether goals for improvement in active function were met over repeated injection cycles. Patients were selected if improved active function (AF) was a primary/secondary goal for at least one cycle of treatment (n = 286/953). They were assessed at the start and end of each cycle using the Goal Attainment T-score alongside the Arm Activity measure (ArmA). Serial changes and predictors of improvement were explored. Across eight cycles, there were 1167 injections for active function, 875/1998 (44%) AF goals were set and 493 (57%) of those goals were achieved. Patients continued to show significant improvement in AF (*p* < 0.001) for each injection at least up to three cycles, which is likely to be clinically significant, especially in patients with milder spasticity who were naïve to BoNT-A. No statistically significant predictors were confirmed on mixed effects modeling. Conclusions: Repeated BoNT-A continued to produce significant improvement in active function for at least three cycles, regardless of patient- or treatment-related factors in this cohort, but type II errors due to small numbers cannot be excluded.

## 1. Introduction

Impairment of motor control is common after stroke or other acquired brain injuries, often resulting in difficulties with coordinated movement. In a large proportion of cases, this is accompanied by disabling spasticity, a disorder arising from damage to the central nervous system and characterized by abnormal, sustained muscle overactivity [1,2]. Clinically, spasticity may manifest as stiffness and pain and, in chronic cases, can lead to complications such as contractures, joint deformities, and pressure ulcers [3]. These consequences can substantially impair functional performance and limit engagement in rehabilitation. While prevalence estimates vary, spasticity has been documented in as many as 42% of stroke survivors, representing approximately 504,000 individuals in the UK [3,4,5,6,7].

Spasticity may affect both bodily function and the execution of activities of daily living. It can hinder ‘active function’, defined as the voluntary use of the affected limb during task performance, as well as ‘passive function’, encompassing the ability to care for the limb and perform essential activities such as dressing and personal hygiene [3,4,8].

Administration of botulinum toxin type A (BoNT-A) has been demonstrated to be a safe and effective localized treatment for reducing spasticity [4,5,6,9,10,11,12,13,14,15,16], and its use is endorsed in routine clinical practice by both national and international clinical guidelines [17,18,19,20,21].

When integrated within a broader rehabilitation program, BoNT-A may improve passive function by reducing muscle overactivity, optimizing joint position, and alleviating strain associated with musculoskeletal deformities [11,22]. Consequently, passive function is frequently identified as a key treatment objective in upper limb spasticity management [11,12]. However, in patients with sufficient residual motor capacity and potential for recovery, treatment priorities may progressively shift towards the restoration of active function [21,22,23,24,25]. In such cases, improvement in motor performance depends primarily on task-specific practice and rehabilitation [26,27,28,29], with BoNT-A serving to reduce spasticity and minimize obstacles to functional recovery [30,31,32].

Goal Attainment Scaling (GAS) provides a structured framework for quantifying the achievement of individualized treatment goals in clinical practice and research [33,34,35,36,37]. Comprehensive guidance on its methodology and application has been described in the literature [33,38,39,40]. The GAS T-score serves as a composite measure of overall goal achievement, whereby increases from baseline indicate clinical progress. A score of 50 (SD ± 10) corresponds to the expected level of attainment, while higher scores reflect outcomes exceeding predefined expectations [8,24]. While GAS demonstrates sensitivity to changes in the management of spasticity, it captures goal attainment rather than objective clinical endpoints and is subject to limitations related to scaling and comparability across patients and practitioners. Consequently, GAS is most appropriately interpreted alongside standardized outcome measures rather than as a standalone assessment tool [24].

The Arm Activity measure (ArmA) was developed to address this need and is a psychometrically robust instrument specifically developed for the assessment of both upper limb active and passive function limitations. It comprises 20 items divided into separate subscales evaluating passive and active functions: a seven-item passive function subscale (ArmA-PF; score range 0–28) and a thirteen-item active function subscale (ArmA-AF; score range 0–52). The measure may be completed by either patients or caregivers. Lower scores on either subscale indicate reduced difficulty in task performance and, therefore, improved functional ability [41]. It also incorporates a third subscale in its latest iteration measuring symptoms and quality of life which was not used in this study.

The Upper Limb International Spasticity (ULIS) program consists of three multinational observational studies designed to characterize and re-evaluate routine clinical practice in the application of BoNT-A for upper limb spasticity management. Central to the programme is the adoption of Goal Attainment Scaling (GAS) as the principal outcome measure, complemented by the use of ArmA.

As a long-term condition, spasticity commonly requires continuous management, and repeated BoNT-A administration has been associated with sustained benefits across a range of clinical symptoms [17,42,43,44]. The third iteration of the ULIS program, ULIS-III, is among the largest multicenter studies to evaluate repeated BoNT-A therapy in upper limb spasticity, including an effectiveness cohort of 953 patients. Within this study, the Upper Limb Spasticity Index (ULSI) was introduced, combining the Focal Spasticity Index with a longitudinal recording of GAS and selected standardized outcome measures aligned with patient-specific treatment priorities at each treatment cycle [45]. The results demonstrated sustained improvements over two years in both overall goal attainment and functional outcomes, spanning passive and active domains [46]. Post hoc analyses further suggested that factors such as the use of injection guidance, female sex, and specific BoNT-A formulations may be predictive of enhanced treatment response [46,47,48].

Building on these findings, the current post hoc analysis examined patients who identified improvement in active upper limb function as a treatment goal. Specifically, the analysis aimed to:

(a) Characterize the active function goals selected by patients and assess goal attainment across repeated treatment cycles over a two-year period;
(b) Evaluate longitudinal changes in active function scores to explore the potential for cumulative functional benefit with repeated treatment;
(c) Investigate whether patient- or treatment-related characteristics were associated with improvements in active function over time.

From a clinical perspective, we hypothesized that patient-related factors associated with treatment improvement or increased injection frequency could include age, sex, type of injury, time since injury, prior exposure to BoNT-A, severity and distribution of spasticity, and the presence of sensory impairment or contractures. Intervention-related predictors were anticipated to comprise the use of injection guidance (electromyogram/stimulation and/or ultrasound) [13,49], the number of muscles targeted, the total dose administered, and the type of guidance technique applied (e.g., electromyography, nerve stimulation, or ultrasound).

## 2. Results

Within the ULIS-III effectiveness population (n = 953), a total of 286 patients (30%) had at least one goal targeting active function (across any cycle of treatment) and were therefore classified as the ‘active function subgroup’ for the purposes of this analysis.

### 2.1. Demographics

The demographics are shown in Table 1.

Those with and without goals related to active function were not significantly different with respect to age, gender, etiology or duration of spasticity. However, as may be expected, the active function subgroup had a lower mean baseline score on the Disability Assessment Scale (DAS) (*p* < 0.001) and also less severe distal spasticity as measured by the composite Modified Ashworth Score (MAS) both proximally and distally (*p* < 0.001). They were also more likely to have had previous injections of BoNT-A (*p* = 0.002). As expected, patients with regional spasticity were less likely to have goals for active function than those with only focal spasticity.

### 2.2. Analysis of Active Function-Related Goals and Achievement Rates

During the 2-year period, there were a total of 1167 injections for the active function subgroup, with a total of 1998 goals, of which 875 (44%) (either primary or secondary goals) were related to active function.

**Figure 1:**
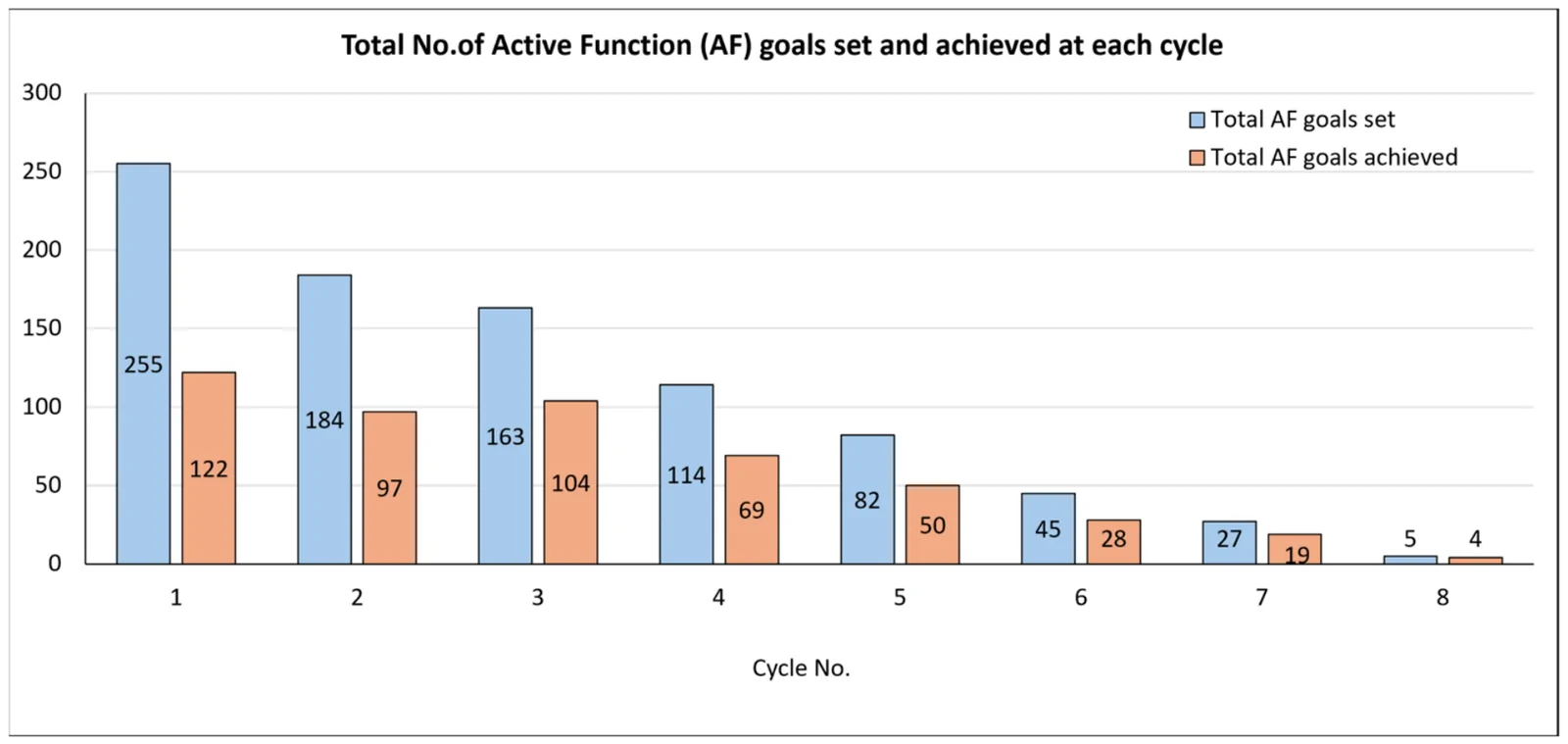
Active function-related goals set and achieved.

The number of active function-related goals decreased across successive cycles to fewer than 30 in cycles 7–8, but only a small number of patients (<50) had that many injections in the 2-year period. The percentage of patients with an active function-related goal remained more constant (ranging from 42 to 47% across seven cycles (see Figure 1 and Table 2)).

**Figure 2:**
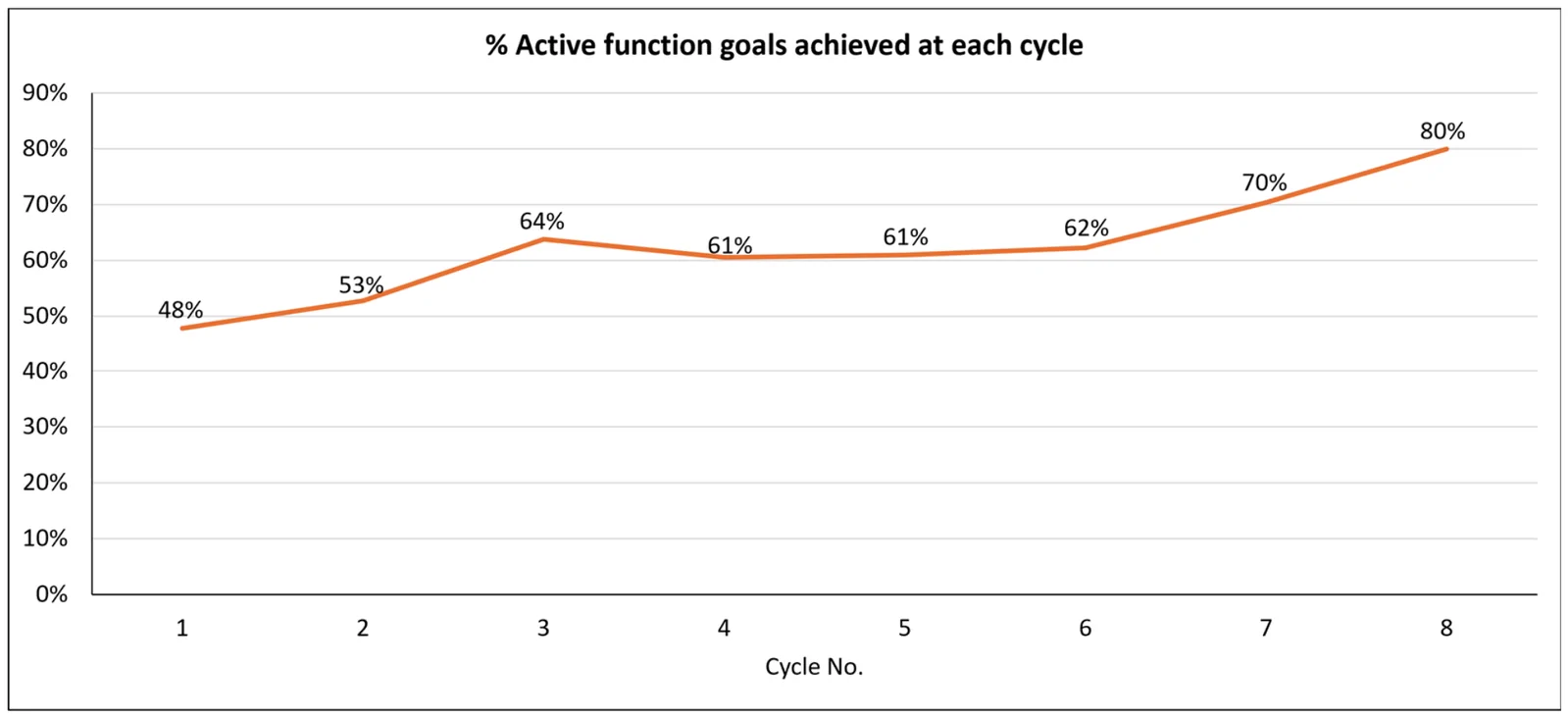
The rate of active function-related goal achievement across the 8 cycles.

The percentage of active function-related goals that were achieved ranged from 48 to 80% across the cycles. In fact, there was a progressive increase in the percentage of active functional goals achieved across the eight cycles (see Figure 2), suggesting that there may be some learning effect regarding what is possible to achieve.

Of active function goals, when sub-classified by body system function, 355 (41%) incorporated grasp and release and 214 (24%) involved holding/stabilizing objects for bimanual function. A total of 176 (20%) goals related to fine dexterity (n = 90) or manipulating objects (n = 86), while 121 (14%) goals related to reaching. The proportion of goal subcategories across the cycles is set out in Table A1 in Appendix A.

### 2.3. Cycle-by-Cycle Analysis of GAS-T and ArmA Active Function Scores

The next step of this evaluation was a cycle-by-cycle analysis (at the population level) of mean goal attainment and ArmA active function scores for all the patients who had a goal related to active function in any cycle, across the eight cycles, and a contemporaneous ArmA active function score. Only three patients had ArmA active function scores for seven or more cycles and so data are only included up to cycle 6.

**Figure 3:**
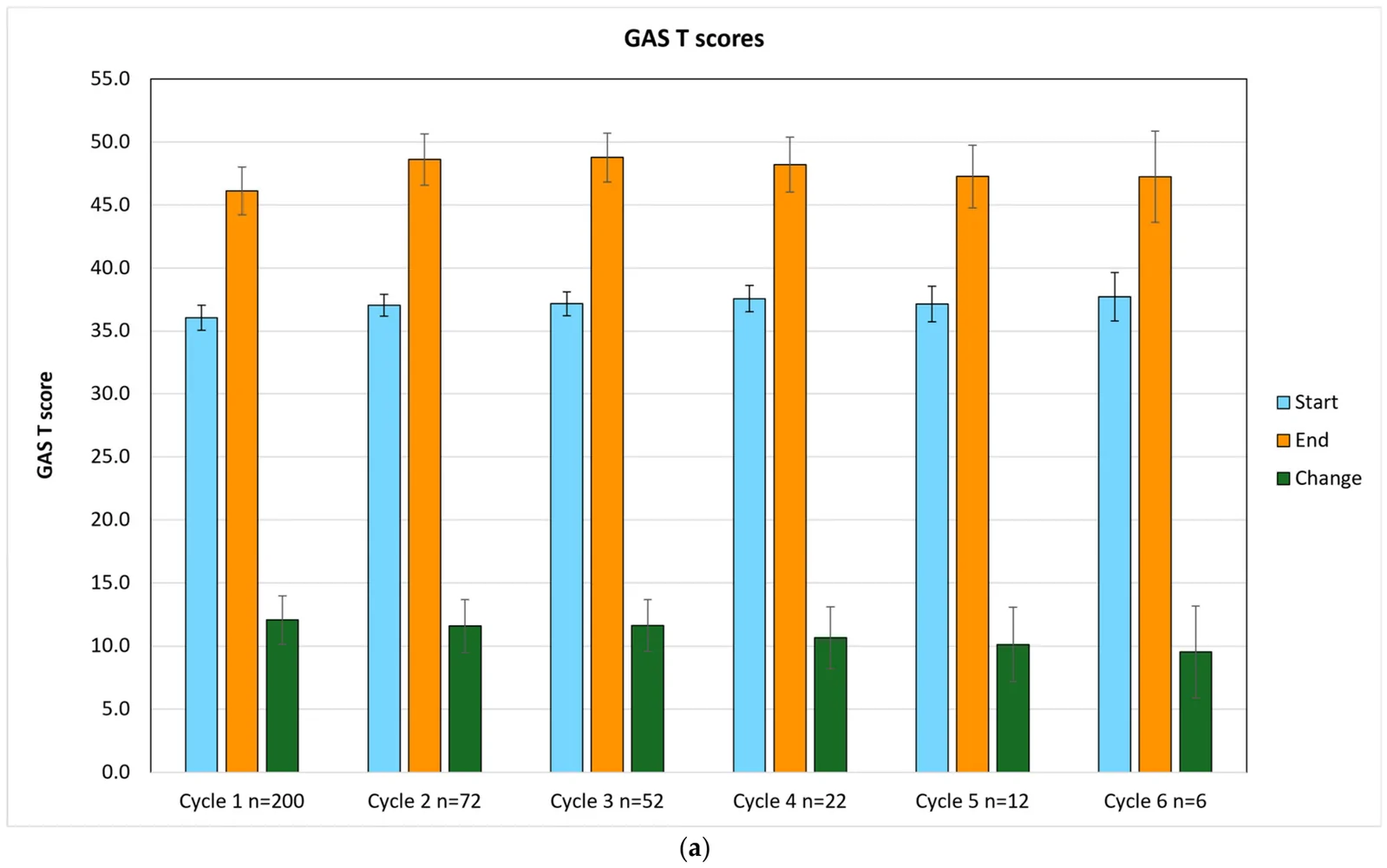

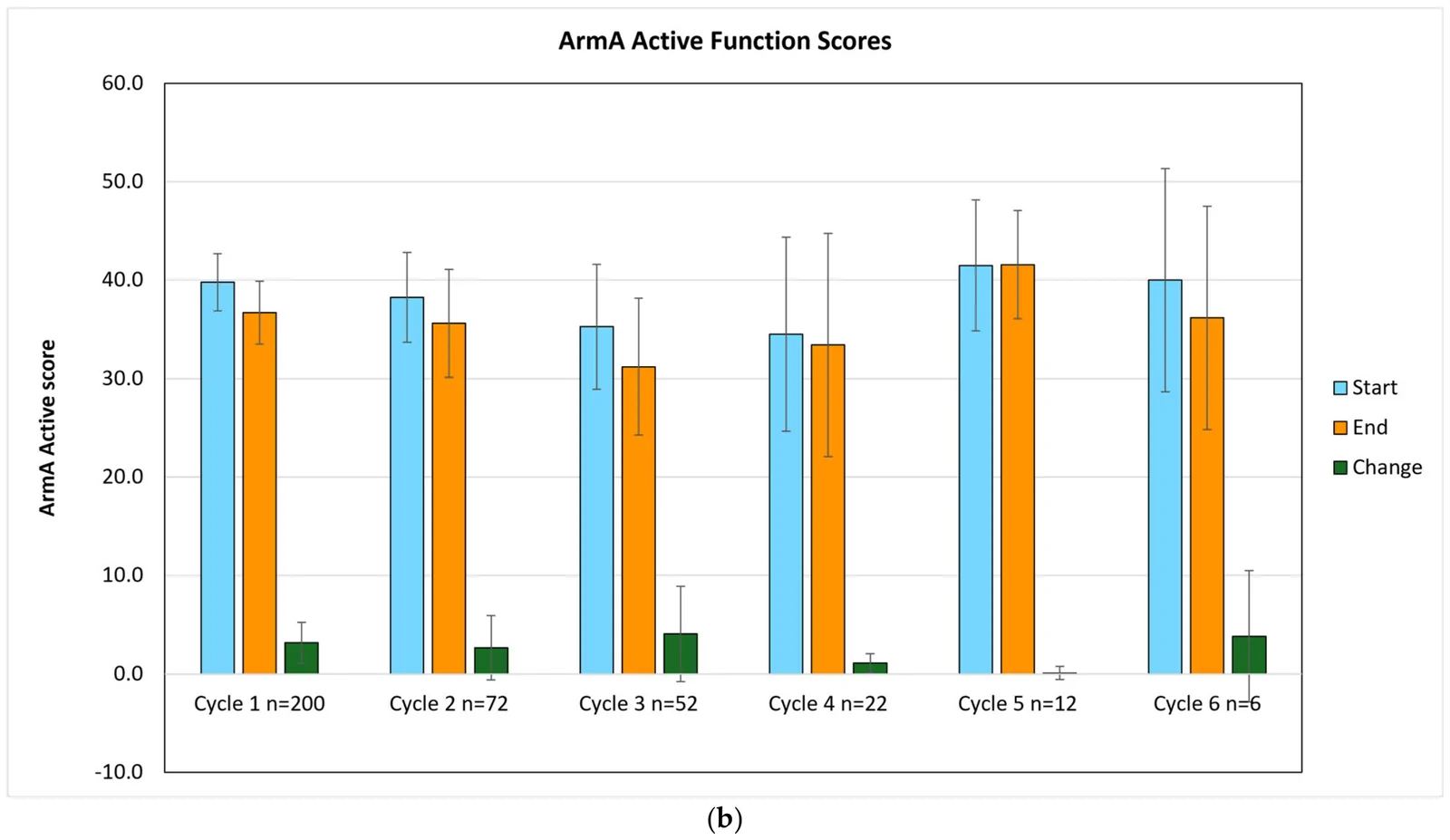
(**a**,**b**): These figures show the mean (95% CI) GAS T-scores and ArmA active function scores for all the patients who had an active function-related goal in any cycle, before and after injection, and the change in scores for each cycle. (**a**) GAS T-scores for overall active function goal attainment across each cycle. Note: GAS = Goal Attainment Scaling. (**b**) ArmA active function scores across each cycle. Note: A reduction in ArmA score is a positive change.

Figure 3 summarizes the mean (95% bootstrapped CI) before and after and the change in scores for (a) the GAS T-score and (b) the ArmA active function for each cycle. (The values and paired *t*-tests are given in Table A2 in Appendix A.)

Figure 3a,b show a significant increase in GAS T-score between the start and end of each cycle (i.e., before and after injection) up to cycle 6 (*p* < 0.001), with change scores ranging from 8.8 to 12.1. There was a significant reduction in ArmA score across the first three cycles (*p* < 0.001), with change scores ranging from 2.7 to 4.2, suggesting that patients may continue to derive overall benefit in terms of active function and goal attainment for at least three cycles of treatment. After three cycles the numbers become too small for statistical analysis. There was no clear trend in the magnitude of change scores across successive cycles.

### 2.4. Serial Changes in Reported Active Function (ArmA) Severity by Number of Cycles

To more directly explore the effects of repeated injection, changes in reported active function across cycles were examined by the number of injection cycles with an ArmA active score recorded. Patients were divided into two ‘cycle frequency’ groups: Group 1 had 1–2 cycles only and Group 2 had 3–4 cycles.

The demographics for these two groups are given in Table A2 in Appendix A. The only significant between-group difference was that Group 2 (i.e., those with more frequent injections) contained more patients with regional spasticity (as opposed to focal spasticity). The trend towards more severe distal spasticity in this group did not reach significance.

**Figure 4:**
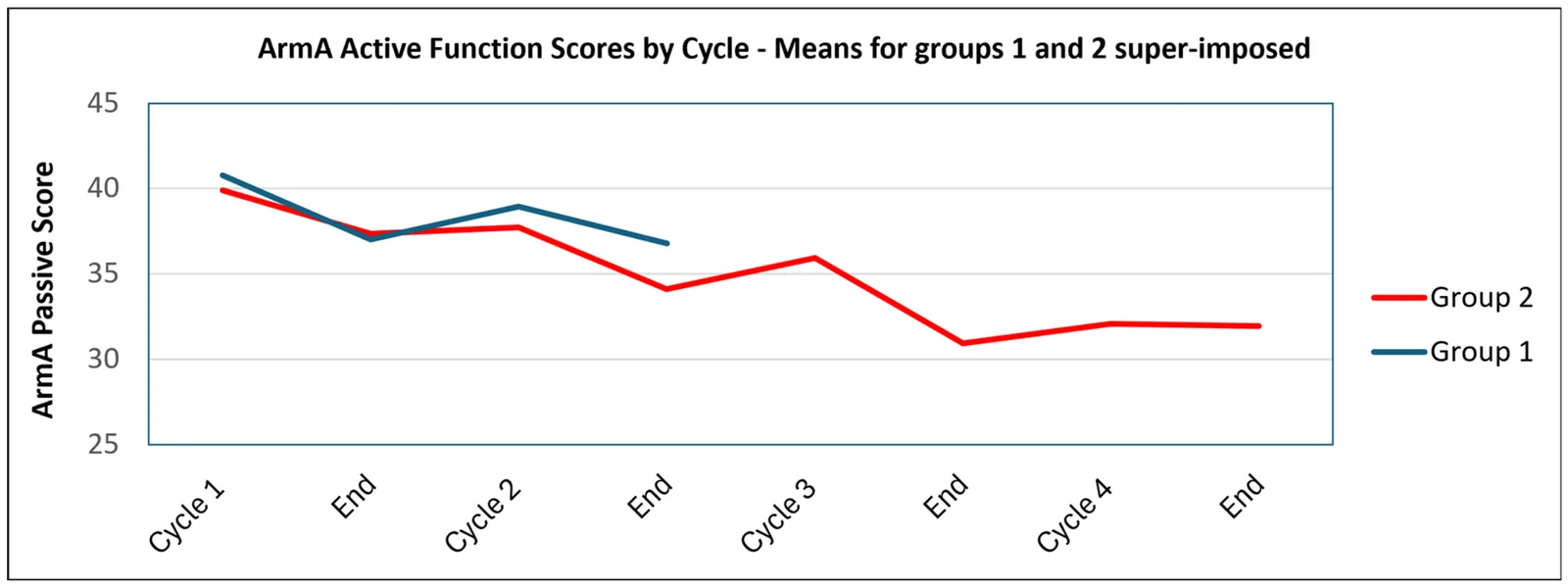
Presents line graphs of the mean ArmA active function scores by cycle (both before and after BoNT-A injection) for the two cycle frequency groups (the mean and 95% CI values are given in Table A3).

On simple visual inspection, the line graphs in Figure 4 suggested a general reduction in ArmA active function scores over successive cycles to cycle 3, both for the start and end cycle scores, suggesting some cumulative effect on improvement in active function. We therefore went on to examine cumulative changes using mixed linear models.

### 2.5. Mixed Linear Models for Cycle and Start Versus End Measurement Effects

Whether the differences between the end and start of the cycle measurements differed in relation to cycles was tested by an interaction term between cycle and measurement time. In both models with different variance–covariance matrix structures for repeated measurements there was no evidence that the difference between end and start of cycle differed in relation to cycle, *p* = 0.40 for an unstructured variance–covariance matrix and *p* = 0.36 for the exponential-in-time model, with the latter favored because of a smaller AIC.

In the main effects models without an interaction term there was no evidence with either model that ArmA scores varied by cycle. Table 3 shows estimates for cycle differences using cycle 1 as the reference. Note that the relevant *p*-value for the effect of the cycle was not important even if individual cycle comparisons appeared to be different, e.g., cycle 3 minus cycle 1.

The confidence intervals for the comparisons were wide in relation to the end of cycle versus start of cycle measurements, as shown below; therefore, due to small number of patients with ArmA active function scores in the individual cycles, a type II error may be present.

However, Table 4 reports the main effect of end versus start after adjustment for cycle in the main effects models. There was strong evidence that ArmA values were lower at the end than at the start by a little over 2.5 points representing an improvement.

### 2.6. Predictors of ArmA Active Function Scores

Because of the small number of patients with ArmA scores in cycle 2, it was difficult to capture predictors of improvement in active function across repeated cycles for the 86 patients who had paired ArmA active function scores between the start of cycle 1 and the end of cycle 3. The mean change in ArmA active function score over this period was 4.7 (SD 9.4; 95% CI 2.7, 6.7. t = 4.6, *p* < 0.001). Univariate analyses of patient- and treatment-related factors are shown in Table A4 and Table A5.

For the univariate associations between the change in ArmA score from the start of cycle 1 to the end of cycle 3 and patient-related characteristics, there was no evidence of an association with age group, acquired brain injury versus not, stroke brain injury versus not, focal versus regional spasticity, severe weakness at baseline, impaired sensation at baseline, contractures present at baseline or time since onset of injury (refer to Table A6 for additional information). There was weak evidence of an association between previous injection with BoNT-A for ULS and the severity of spasticity at baseline (especially proximal spasticity) as measured by the composite MAS. However, none of these associations were evident when tested with mixed effects models.

With respect to treatment-related characteristics, there was no evidence of an association with use of injection guidance, the number of muscles injected, or the dose of BoNT-A used. In addition, there were no significant differences between any of the three BoNT-A agents used.

## 3. Discussion

The present analysis provides further insight into the long-term management of active upper limb dysfunction in patients receiving repeated BoNT-A treatment within routine clinical practice over a 2-year period. Among the 953 patients enrolled in ULIS-III, approximately one-third (286 patients) identified active function as a treatment goal at some point during their treatment. These patients continued to show a significant response in terms of both goal attainment up to at least seven injection cycles and in terms of improvement in active function (as measured by the Arm active function score) up to three cycles. Interpretation of outcomes beyond cycle 3 should be approached with caution because the number of participants decreased substantially with subsequent cycles, increasing the possibility of a type II error.

Despite the gradual reduction in the number of active function goals set, the percentage of goals related to active function remained roughly similar (42–47%) across the first seven cycles. Moreover, goal attainment rates increased across successive treatment cycles. This may suggest a refinement of goal setting and treatment expectations over time, leading to better alignment between therapeutic objectives and achievable outcomes. Yet, decreasing patient numbers across treatment cycles limits the strength of these conclusions regarding long-term effects and should be considered when interpreting these findings.

### 3.1. Response to Repeated Injections

Overall goal attainment scores changed from the start to the end of each cycle by ≥10 (the minimally important clinical difference) in seven cycles.

Clinically significant improvement in active function as measured by the ArmA was seen between the start and end of each cycle (i.e., before and after injection), which remained significant across the first three cycles, confirming that patients continued to derive overall benefits in active function for at least three cycles of treatment [50,51]. Beyond cycle 4, the numbers become too small for statistical analysis, but the mean change from the start of cycle 1 to the end of cycle 3 was 4.7 (95% CI 2.7, 6.7) which is well within the range for a minimally important clinical difference for ArmA (1.1–2.5) [52].

Goals for active function are primarily focused on using the affected upper limb in tasks involving grasping and releasing objects, holding/stabilizing an object for in bimanual function, fine dexterity or manipulation of objects, reaching, and occasionally lifting and carrying. All of these elements of body function featured to a greater or less extent in the goals set in this evaluation [53,54].

The total intervention is often complex with injection of botulinum toxin only being part of a number of interventions provided [55,56]. Other elements of intervention are likely to include strengthening and task-practice as components of physical therapy over time [23]. As such, the expectation will be that repeat injection is likely to be required to maintain the benefits initially achieved.

### 3.2. Serial Changes in Severity

Overall, in the two groups, Group 1 (1–2 cycles only) and Group 2 (3–4 cycles only) which were used to further explore serial change, a trend towards an average reduction in ArmA active function over successive cycles was identified (both at the start of the new cycle and again at the end of that cycle), with reductions in absolute values visible at the start and end of each cycle. This serial reduction suggests that there might be some cumulative effect on improvement in active function. While our analysis may not take into account all possible covariates, it is likely that over time, even though spasticity increases again as the botulinum toxin effect reduces, active function may become easier. The potential mechanism of action for this change is in both the potential stabilization of spasticity over time [57] and the effect of interventions such as task-practice as well as other interventions, such as splinting and positioning, providing an ongoing stretch of muscle and other structures acting across the joints involved to maintain range of movement and ease of use of the limb. For the multiple tests involved, *p*-values were adjusted, using the Holm–Bonferroni method, on a case-by-case basis for each analysis.

### 3.3. Patient-Related Predictors of Active Function Improvement and the Frequency of Injection

As noted, goals incorporating active function are primarily focused on using the affected upper limb for activities which have task components incorporating grasping and releasing objects, holding/stabilizing an object for use in bimanual function, fine dexterity or manipulation of objects, reaching, and occasionally lifting and carrying [9,26,35]. However, these components on their own are not sufficient to complete an activity. For example, the activity might be to cook a meal, and another activity might be then to eat that meal. There are multiple complex components to these activities, which have multiple task elements within them, which are not all about motor control or motor planning [23,30,58]. Taking the motor planning element alone, this is still much more complex than only manipulation of objects or reaching. This complexity, and the need for training these different elements once spasticity has been reduced, is likely to be a major reason why active function improvement is seen in a smaller subgroup of patients undergoing spasticity treatment [59,60,61,62].

In our cohort there is also the risk that the failure to demonstrate associations and trends at a statistical level may represent a type II error, where changes are small, but have occurred, or (most plausible) changes are occurring but emerge gradually with successive treatment and continued task-practice of rehabilitation-defined practice tasks for active function improvement.

### 3.4. Comparison of Findings with Other Studies

There are clear precedents for training of motor function in general, which also emphasize the complexity and dosages of training needed [59,60]. In our study, in real-life routine practice, these elements were not considered and are known to be variable in clinical rehabilitation settings. There are, however, good examples of incorporation of task training into spasticity management using botulinum toxin injection as a component of management [23,30,35,58,63,64]; these approaches need to be further incorporated into routine practice to optimize active function goal attainment and functional progression.

### 3.5. Important Messages for Clinicians Managing Upper Limb Spasticity

Measurable improvements in active function were achieved with repeated injections of BoNT-A up to at least three cycles.

Management of spasticity with the goal of active function improvement needs to incorporate task training to maximize optimal goal attainment.

### 3.6. Strengths and Limitations

The authors acknowledge a number of strengths and limitations of the overall study.

1. Strengths include the large sample size, taken from real-life clinical practice with broad international representation, including with multiple etiologies and all BoNT-A products, giving generalizability to the findings.
2. However, although there were over 1000 patients enrolled in the overall study, less than a third of the patients had a goal for improvement in active function, and the number of AF goals set decreased over successive cycles, becoming too small for analysis. The failure to demonstrate associations and trends at a statistical level may well represent a type II error due to small numbers and a larger study would be required to confirm the absence of association.
3. The observational design introduces inherent limitations of the study. The absence of a control group precludes causal inference, while variation in site participation across countries may affect representativeness. Potential sources of bias include differences in clinician expertise, injector practices, treatment decision-making, and external prescribing restrictions that may influence the frequency of treatment.
4. Given the observational design, the reasons for treatment discontinuation are unknown, which may represent a degree of attrition bias. Patients who experienced greater clinical benefit may have been more likely to continue treatment and remain available for follow-up. While our findings suggest continued goal attainment among patients who remain on treatment, they do not definitively demonstrate a cumulative functional benefit of repeated BoNT-A injection. Further studies are needed to validate this observation.
5. The ULIS-III study was conducted in real-life practice and spasticity-related limitation in active function may be multifactorial.
6. The ULIS-III study has provided a large and valuable dataset for post hoc analyses. The greater the extent of the post hoc analysis, the more risk there is of introducing statistical error through multiple tests. In this study, we have explored in some detail the impact of patient- and treatment-related characteristics, but there may be other characteristics that have not yet been explored

## 4. Conclusions

The limitations in these analyses are acknowledged. However, findings demonstrate that improvement in active function is a clear goal for treatment with BoNT-A in arm spasticity and that repeated injections over a 2-year period continue to produce significant improvement in active function over at least three cycles regardless of factors such as age, etiology of injury, time since onset, distribution of spasticity, or factors such as dose or BoNT-A agent. Importantly, improvement in active function should be viewed within the context of a comprehensive rehabilitation program, in which BoNT-A serves as one component of a broader strategy to reduce spasticity and facilitate functional recovery [16].

## 5. Materials and Methods

### 5.1. Study Design and Participants

This is a retrospective secondary analysis of data collected from the ULIS-III study (NCT02454803). Full details of the methodology have been previously described [46,65].

In brief, ULIS-III was an international observational, prospective, longitudinal cohort study following patients with upper limb spasticity over 2 years, treated through integrated programs incorporating BoNT-A injections and physical management as delivered in real-life clinical practice. It was conducted in 58 centers, across 14 countries and four continents.

Specialist centers recruited up to 30 consecutive patients (≥18 years old) in whom a decision had been made to treat upper limb spasticity with BoNT-A. Patients received any licensed BoNT-A formulation according to local regulatory approval and routine practice.

Treatment was goal-directed, and patients could change their primary and/or secondary goals at the start of each treatment cycle. Ethical approval and written informed consent for the collection of anonymized data were obtained where required.

### 5.2. Outcome Assessment and Measures

Patients were assessed at baseline and follow-up visits throughout the study using the Upper Limb Spasticity Index (ULSI). This records GAS alongside a limited set of standardized measures, selected specifically according to the priority treatment goal area(s) for each cycle.

Treatment goals were captured using the Goal Attainment Scaling Evaluation of Outcome for Upper Limb Spasticity (GAS-eous) Tool [65]. Up to three goals (one primary and two secondary) could be set per cycle. Goal attainment was converted to a GAS T-score using the GAS-light method. The GAS-T formula is designed such that, if goals are achieved as expected, the mean GAS T-score will be 50 (SD +/− 10) [39]. A change of 10 points is considered the minimal clinically important difference in spasticity management [33].

Of the 953 patients in the effectiveness population, this secondary analysis focused on those patients who identified active function as a treatment goal during at least one study cycle. The standardized measure for active function was ArmA [52,66].

### 5.3. Statistical Analysis

Patients were included in the ‘active function subgroup’ if they had at least one active function goal (primary or secondary) during any treatment cycle. A cycle is defined as the before- and after-assessment periods of each injection of BoNT-A, resulting in two visits per cycle.

Because ULIS-III was an observational cohort study reflecting real-life practice, active function-related goals and corresponding ArmA assessments were not necessarily recorded at every cycle, creating challenges for longitudinal analysis. An initial exploratory descriptive and visual analysis was performed/undertaken to examine trends in the data before applying more sophisticated statistical analysis.

Previous analyses have demonstrated that patients received between one and eight cycles during the study. However, very few patients with active function goals had 7–8 cycles (n = 3 and n = 2 respectively), so analyses were limited to the first four cycles. To explore any differences between patients who required ‘more frequent’ versus ‘less frequent’ injections for active function-related goals, patients were stratified based on the maximum number of injections received (cycle frequency) during the 2-year period: Group 1 only had 1–2 cycles and Group 2 had 3–4 cycles.

No outliers were removed nor were any transformations made to the original variables. Missing data were not imputed and *p*-values in this study were adjusted to control for multiple comparisons within each set of analyses, using the Holm–Bonferroni method [67]. Analysis of the data was completed in SAS (version 9.4).

The primary aim of this study was to evaluate changes in GAS T-scores and ArmA active function scores over repeated treatment cycles in patients whose treatment goals included the improvement of active function. Mean scores before and after each cycle and change scores for each cycle were calculated with 95% bootstrapped confidence intervals (CIs) based on 1000 samples and were compared using paired *t*-tests, stratified by cycle frequency group.

Two modeling approaches were used to identify univariate associations between ArmA active function scores and potential predictors of these. First, the difference between the end of treatment assessment in cycle 3 and the start of treatment assessment in cycle 1 (as an overall baseline measurement) was directly modeled using *t*-tests, linear regression and correlation coefficients. Second, mixed linear modeling was used to account for repeated measurements across cycles. Associations for categorical predictors were expressed as differences in ArmA scores between categories while continuous predictors were expressed as regression coefficients per unit increase.

A mixed effects model was used to examine variables that predicted ArmA active function scores (at the start and end of each cycle, separately) while controlling for their confounding effect. The observations represented participants and cycle number, as opposed to participants alone. Because this study had an AB design, with a maximum of six cycles, the observations in the data do not fulfil the independence assumption. We controlled for dependency between observations over time by including a random effect term for each individual. Using a model by cycle and person provided more power in detecting significant differences between the predictor variables and response variables in this study [68].

Based on experience from previous analyses, candidate predictive patient-related factors for active function reduction might include age, gender, chronicity of spasticity, previous treatment with BoNT-A, severity and distribution of spasticity (as measured by the composite MAS for the proximal (elbow and shoulder) [46] and distal (hand, wrist and finger) muscles, and the total number of treatment cycles. We also hypothesized that intervention-related factors may include the number of muscles injected, anatomical distribution of injections, the total dose and the use of injection guidance techniques (e.g., electromyography, nerve stimulation, or ultrasound). Because different preparations of BoNT-A were used (Abobotulinum Toxin, Incobotulinum Toxin, and Onabotulinum Toxin), which have different units, the total dose used was expressed as a proportion of the maximum recommended dose for each formulation.

## Figures and Tables

**Table 1:** Demographics of the overall effectiveness population and the active function subgroup.

		Patients with:			
	Effectiveness Population	Active Function Goals	No Active Function Goals	Odds Ratio 95% CI	*p*
	N/953 (%)	N/286 (%)	N/666 (%)		
**Demographic**					
**Sex**				1.11 (0.84, 1.5)	0.478
Male	537 (56)	166 (58)	370 (56)		
Female	416 (44)	120 (42)	296 (44)		
**Age**				0.57 (0.42, 0.77)	<0.001
≤60 years	617 (65)	210 (73)	407 (61)		
>60 years	335 (35)	76 (27)	259 (39)		
**Type of injury**				0.70 (0.44, 1.11) *	0.129
Acquired brain injury	870 (91)	255 (80)	614 (92)		
Spinal cord injury	15 (2)	8 (3)	7 (1)		
Congenital	44 (5)	18 (6)	26 (4)		
Progressive neurological	20 (2)	4 (1)	16 (3)		
Other	4 (<1)	1 (<1)	3 (1)		
**Etiology of acquired brain injury**				0.64 (0.45, 0.90) **	0.010
Vascular (stroke)	786 (90)	222 (78)	563 (85)		
Trauma	71 (8)	30 (11)	41 (56)		
Hypoxic	25 (3)	4 (1)	21 (3)		
Tumour	19 (2)	9 (11)	10 (6)		
Inflammatory/ infective/other	52 (6)	3 (1)	12 (2)		
**Previous injections with BoNT-A for arm spasticity**				1.10 (0.82, 1.48)	0.525
No (naïve)	318 (33)	91(32)	226 (34)		
Yes (non-naïve)	635 (67)	195 (68)	440 (66)		
**Distribution of spasticity**				0.60 (0.43, 0.83)	0. 002
Focal	190 (20)	74 (26)	115 (17)		
Regional	763 (80)	212 (74)	551 (83)		
Total	953	286 (30)	666 (70)		
**Time since onset of injury**				*t*-test	
Years—mean (95% CI)	7.6 (7.0, 8.2)	7.8 (6.5, 9.0)	7.5 (6.7, 8.3)	t = −0.41	0.686
**Baseline function/spasticity—Mean (95% CI)**					
Baseline DAS total score	6.1 (5.9, 6.3)	5.2 (4.9, 5.1.)	6.5 (6.3, 6.7)	t = 7.0	<0.001
Proximal composite MAS	3.7 (3.6,3.8)	3.4 (3.2, 3.6)	3.8 (3.7, 3.9)	t = 4.0	<0.001
Distal composite MAS	6.1 (5.9, 6.2)	5.4 (5.1, 5.7)	6.4 (6.2, 6.6)	t = 5.9	<0.001

BoNT-A = botulinum toxin A; MAS = Modified Ashworth Scale; DAS = Disability Assessment Scale; CI = bootstrapped confidence interval. χ^2^ = Chi square. * Adjusted *p*-values for the difference between the subgroups of the effectiveness population with one or more active function-related goal (n = 286) and with no pain-related goals (n = 667). ** Levene’s test was statistically significant.

**Table 2:** Analysis of active function-related goals and achievement rates.

Cycle	Total N in Sample	Total N of Goals Set	N (%) * Goals Related to Active Function (AF)	N (%) * Active Function Goals Achieved
1	286	546	255 (47)	122 (48)
2	254	443	184 (42)	97 (53)
3	221	378	163 (43)	104 (64)
4	172	272	114 (42)	69 (61)
5	115	177	82 (46)	50 (61)
6	68	98	45 (46)	28 (62)
7	43	58	27 (47)	19 (70)
8	8	16	5 (31)	4 (80)
Total	1167	1988	875 (44)	493 (57)

* % refers to the denominator in the same row from the previous column.

**Table 3:** Mixed effects models for cycle for variation in ArmA scores by cycle over 4 cycles.

**Model 1: Unstructured Variance Covariance Matrix**		***p*** **-value**
**Overall: For effect of cycle**		**0.20**
	**Estimate (95% CI)**	
Cycle 2 minus 1	−1.55 (−3.77 to 0.66)	0.17
Cycle 3 minus 1	−2.54 (−4.95 to −0.13)	0.039
Cycle 4 minus 1	−1.08 (−3.72 to 1.57)	0.42
**Model 2: Exponential-in-time variance covariance matrix**		***p*** **-value**
**Overall: For effect of cycle**		**0.23**
	**Estimate (95% CI)**	
Cycle 2 minus 1	−1.75 (−4.00 to 0.50)	0.13
Cycle 3 minus 1	−2.31 (−4.76 to 0.13)	0.064
Cycle 4 minus 1	−1.13 (−3.78 to 1.52)	0.40

**Table 4:** Mixed effects models for cycle for variation in ArmA scores without an interaction term.

	End Minus Start Estimate (95% CI)	*p*-Value
Model 1: Unstructured variance covariance matrix	−2.88 (−3.66, −2.10)	<0.0001
Model 2: Exponential-in-time variance covariance matrix	−2.68 (−3.44, −1.93)	<0.0001

## Data Availability

Data-sharing is restricted due to the limits of patient confidentiality and consent. Further details on Ipsen’s sharing criteria, eligible studies, and process for sharing are available here: https://vivli.org/members/ourmembers/ (accessed on 29 September 2026). Any requests should be submitted to www.vivli.org for assessment by an independent scientific review board. This study did not produce patient level data but was a secondary analysis of the ULIS III data.

## Appendix A. Additional Tables

**Table A1:** The number and percentage of active function goal subcategories across the cycles.

Goal Subcategory	Cycle 1 (%)	Cycle 2 (%)	Cycle 3 (%)	Cycle 4(%)	Cycle 5 (%)	Cycle 6 (%)	Cycle 7(%)	Cycle 8 (%)	Total No. (%)
Reaching	41 (16)	24 (13)	17 (10)	9 (8)	16 (20)	9 (20)	5 (19)	0 (0)	121 (14)
Grasp/release/grip	102 (40)	69 (38)	73 (45)	48 (42)	27 (33)	17 (38)	14 (52)	5(100)	355 (41)
Holding/bimanual function	56 (22)	49 (27)	42 (26)	28 (25)	24 (29)	11 (24)	4 (15)	0 (0)	214 (24)
Manipulating objects	23 (9)	18 (10)	17 (10)	13 (11)	8 (10)	4 (9)	3 (11)	0 (0)	86 (10)
Dexterity/fine motor	27 (11)	23 (13)	14 (9)	16 (14)	6 (7)	3 (7)	1 (4)	0 (0)	90 (10)
Lifting/carrying	5 (2)	1 (1)	0 (0)	0 (0)	1 (1)	1 (2)	0 (0)	0 (0)	8 (1)
Total N	255	184	163	114	82	45	27	5	875

**Table A2:** GAS T-scores and ArmA scores across the cycles for patients with an active function goal in that cycle.

		Start of Cycle				End of Cycle				Change				Paired *t*-Tests		
				95% CI				95% CI				95% CI				One-Sided
Cycle	N=	Mean	(SD)	Lower	Upper	Mean	(SD)	Lower,	Upper	Mean	(SD)	Lower	Upper	*t*	*df*	*p*-Value
**GAS T-scores**																
1	200	35.6	(4.2)	35.0,	36.2	47.6	(8.2)	46.5,	48.7	12.0	(8.2)	10.9,	13.2	−11.6	199	<0.001
2	72	36.9	(3.7)	36.0,	37.7	47.4	(8.5)	45.4,	49.4	10.5	(8.9)	8.4,	12.6	−10.6	71	<0.001
3	52	36.8	(3.4)	35.8,	37.7	48.0	(6.3)	46.3,	49.8	11.3	(6.2)	9.6,	13.0	−8.1	51	<0.001
4	22	36.3	(4.6)	34.4,	38.3	46.5	(11.2)	41.8,	51.2	10.1	(10.5)	5.7,	14.5	−9.4	21	<0.001
5	12	37.2	(3.6)	35.2,	39.3	46.1	(7.4)	41.9,	50.2	8.8	(7.0)	4.8,	12.8	−8.3	11	<0.001
6	6	35.0	(5.1)	30.9,	39.1	45.0	(9.6)	37.3,	52.6	10.0	(6.8)	4.5,	15.4	−11.0	14	<0.001
**ArmA active scores**																
1	200	39.8	(10.5)	38.4,	41.3	36.7	(11.2)	35.1,	38.2	3.2	(7.3)	2.1,	4.2	6.1	199	<0.001
2	72	38.3	(9.5)	36.1,	40.5	35.6	(10.9)	33.1,	38.1	2.7	(6.9)	1.0,	4.3	3.2	71	<0.001
3	52	35.3	(11.8)	32.1,	38.5	31.2	(13.0)	27.7,	34.7	4.1	(8.9)	1.6,	6.5	3.3	51	<0.001
4	22	34.5	(12.8)	29.2,	39.8	33.4	(14.1)	27.5,	39.3	1.1	(7.9)	−2.4,	4.6	0.6	21	0.262
5	12	41.5	(6.3)	37.9,	45.1	41.6	(5.2)	38.7,	44.5	−0.1	(3.6)	−2.4,	2.2	0.1	11	0.469
6	6	40.0	(8.1)	33.5,	46.5	36.2	(7.6)	30.1,	42.2	3.8	(4.7	−1.1,	8.7	2.0	14	0.050

*t* = *t*-test statistic; *df* = degrees of freedom; *p* = *p*-value; GAS = Goal Attainment Scaling.

**Table A3:** Demographics for each of the three cycle frequency groups.

	Group 1 N = 82 (%)		Group 2 N = 100 (%)		Odds Ratio (95% CI)	*p*-Value
**Demographic**						
**Sex—n (%)**					1.15 (0.6, 2.1)	0.654
Male	49	(60)	63	(63)		
Female	33	(40)	37	(37)		
**Age—n (%)**						
≤60 years	57	(70)	74	(74)	0.80 (0.41, 1.5)	0.502
>60	25	(30)	26	(26)		
**Type of injury—n (%)**						
Acquired brain injury	71	(87)	95	(95)	2.9	0.460
Non-brain injury	11	(13)	5	(5)	1.0, 8.9	
**Etiology of acquired brain injury—n (%)**						0.222
Vascular (stroke)	63	(77)	84	(84)	1.6	
Non-vascular	19	(23)	16	(16)	(0.76, 3.32)	
**Previous injections with BoNT-A for upper limb spasticity—n (%)**						0.063
No (Naïve)	40	(49)	35	(35)	1.77 (0.97, 3.2)	
Yes (Non-Naïve)	42	(51)	65	(65)		
**Distribution of spasticity—n (%)**						
Focal	26	(32)	18	(18)	2.1 (1.1, 4.2)	0.032
Regional	56	(68)	82	(82)		
**Baseline spasticity scores**	Mean	95% CI	Mean	95% CI	*t*-test	
Proximal composite MAS	3.3	(2.9, 3.6)	3.6	(3.3, 3.8)	−1.24	0.212
Distal composite MAS	4.8	(4.2, 5.4)	5.5	(5.0, 6.0)	−1.94	0.055
**Time since onset of injury**						
Years—Mean (95% CI)	7.5	(5.0, 9.9)	6.6	(5.0, 8.1)	0.611	0.271

CI = confidence interval, MAS = Modified Ashworth Scale; *p*-value—Pearson Chi square.

**Table A4:** Mean GAS T-scores and Arma active function scores by cycle frequency group with 95% bootstrapped CIs.

		Group 1 (n = 82)					Group 2 (n = 100)					
	Before			After			Before			After		
Cycle	N	Mean (SD)	95% CI	N	Mean (SD)	95% CI	N	Mean (SD)	95% CI	N	Mean (SD)	95% CI
**GAS T-scores**												
1	82	35.6 (4.5)	34.6, 36.5	79	46.9 (9.0)	44.8, 48.9	100	36.4 (3.5)	35.7, 37.1	100	48.9 (8.3)	47.3, 50.4
2	50	37.8 (3.0)	36.8, 38.6	37	47.6 (8.8)	44.8, 50.3	100	37.6 (3.4)	36.9, 38.2	99	49.4 (8.0)	47.9, 51.0
3							100	37.8 (2.9)	37.2, 38.3	100	50.2 (6.3)	49.0, 51.4
4							63	37.7 (3.10)	36.9, 38.4	54	50.1 (5.7)	48.7,51.6
**Arma active scores**												
1	72	40.8 (11.4)	37.9, 43.4	67	37.0 (11.8)	34.0, 39.6	63	39.9 (10.4)	37.3, 42.4	60	37.4 (10.9)	34.7, 40.0
2	16	38.9 (12.7)	32.1, 44.5	20	36.8 (12.2)	31.3, 42.1	32	37.7 (9.3)	34.4, 40.8	49	34.1 (11.0)	31.0, 37.5
3							36	35.9 (12.4)	31.8, 39.7	54	30.9 (12.7)	27.7, 34.4
4							14	32.1 (13.8)	24.8, 38.9	26	32.0 (13.5)	26.6, 37.0

**Table A5:** Within-group comparisons of ARMA active scores for 86 patients completing three cycles of ULIS: dichotomous variables.

		Change in ARMA Active Score (Δ = End Cycle 3—Start Cycle 1) (N = 86)			Model 1: Unstructured Variance Covariance Matrix			Model 2: Exponential-in-Time Variance Covariance Matrix		
Variable	N=	Mean (SD)	Difference (95% CI)	*p*	Least Squares Mean (SE)	Difference (95% CI)	*p*	Least Squares Mean (SE)	Difference (95% CI)	*p*
**PATIENT CHARACTERISTICS**										
**Gender**						Overall:	0.35		Overall:	0.34
Male	50	−6.32 (9.56)	3.90 (−0.10, 7.91)	0.056	−6.59 (1.67)	−2.19 (−7.29, 2.91)	0.40	−6.16 (1.70)	−2.26 (−7.9, 2.88)	0.39
Female	36	−2.42 (8.71)			−4.41 (1.98)			−3.91 (1.96)		
**Age Group**										
<60 year	66	−4.38 (8.11)	−1.32 (−6.10, 3.45)		−5.29 (1.46)	−1.92 (−7.86, 4.03)	0.53	−5.0 (1.46)	−1.07 (−7.18, 5.02)	0.73
60+	20	−5.7 (12.9)		0.58	−7.21 (2.65)			−6.08 (2.73)		
**Previous BoNT-A**						Overall:	0.94		Overall:	0.91
Yes	68	−3.56 (9.52)	5.39 (0.56, 10.2)	0.029	−4.41 (1.49)	5.63 (−0.09, 11.4)	0.054	−4.19 (1.50)	4.57 (−1.26, 10.4)	0.12
No	18	−8.94 (7.59))			−10.0 (2.50)			−8.75 (2.55)		
**ABI/Other**						Overall:	0.64		Overall:	0.67
ABI	78	−4.87 (9.61)	−2.0 (−8.94, 4.94)	0.57	−6.13 (1.34)	−4.70 (−13.6, 4.17)	0.30	−5.60 (1.36)	−4.17 (−13.0, 4.65)	0.35
Not ABI	8	−2.88 (6.71)			−1.43 (4.30)			−1.43 (4.27)		
**Etiology**						Overall:	0.73		Overall:	0.69
Vascular (stroke)	66	−4.74 (10.3)	0.24 (−4.54, 5.03)	0.92	−7.09 (1.44)	−5.89 (−11.9 to 0.16)	0.056	−6.33 (1.46)	−4.66 (−10.8, 1.48)	0.14
Not vascular	20	−4.50 (5.74)			−1.20 (2.72)			−1.67 (2.76)		
**Focal/Regional**						Overall:	0.17		Overall:	0.28
Regional ULS	58	−5.03 (9.49)	−1.07 (−5.38, 3.24)	0.62	−6.22 (1.49)	−1.52 (−7.18, 4.16)	0.60	−5.64 (1.50)	−1.27 (−7.0, 4.43)	0.66
Focal ULS	28	−3.96 (9.24)			−4.70 (2.47)			−4.37 (2.47)		
**Baseline Weakness**						Overall:	0.68		Overall:	0.69
Severe	11	−1.00 (5.22)	4.08 (−1.89, 10.1)	0.18	−0.71 (2.89)	5.49 (−0.78, 11.8)	0.086	−0.71 (2.86)	5.02 (−1.19 to 11.2)	0.11
Not	74	−5.08 (9.72)			−6.21 (1.33)			−5.74 (1.34)		
**Impaired Sensation**						Overall:	0.33		Overall:	0.54
Yes	42	−3.14 (9.24)	2.78 (−1.22, 6.80)	0.17	−4.87 (1.86)	1.15 (−3.92, 6.22)	0.65	−4.72 (1.88)	0.51 (−4.60, 5.63)	0.84
No	43	−5.93 (9.34)			−3.04 (1.78)			−5.23 (1.80)		
**Contractures**						Overall:	0.31		Overall:	0.16
Yes	61	−3.93 (10.3)	−2.19 (2.28, 6.67)	0.33	−4.75 (1.46)	3.14 (−2.51, 8.79)	0.28	−4.38 (1.48)	2.62 (−3.11, 8.35)	0.37
No	24	−6.13 (6.29)			−7.89 (2.47)			−7.0 (2.51)		
TREATMENT CHARACTERISTICS										
**Injection Guidance Cycle 1**						Overall:	0.56		Overall:	0.66
Yes	77	−4.68 (9.35)	−0.57 (−6.42, 7.57)	0.87	−4.51 (1.43)	6.28 (−0.55, 13.1)	0.071	−4.52 (1.42)	1.95 (−5.35, 9.25)	0.60
No	8	−5.25 (10.7)			−10.8 (3.16)			−6.47 (3.42)		
**Injection Guidance Used in Center**						Overall:	0.25		Overall:	0.79
Yes		−4.36 (9.26)	5.64 (−2.91, 14.2)	0.19	−4.62 (1.36)	10.7 (2.62, 18.7)	0.01	−4.63 (1.37)	4.79 (−4.51, 14.1)	0.31
No		−10.0 (10.7)			−15.3 (3.86)			−9.42 (4.51)		

**Table A6:** Within-group comparisons of ARMA active scores for 86 patients completing three cycles of ULIS: continuous variables.

		Change in ARMA Active Score (Δ = End Cycle 3 − Start Cycle 1) (N = 86)				Model 1 Unstructured Variance Covariance Matrix			Model 2: Exponential-in-Time Variance Covariance Matrix		
Variable	N=	Pearson’s Correlation	Slope	(95% CI)	*p*-Value	Slope	(95% CI)	*p* Value	Slope	(95% CI)	*p* Value
**PATIENT CHARACTERISTICS**											
Spasticity at baseline:						Slope	(95% CI)	0.35	Slope	(95% CI)	
MAS total score	81	−0.24	−0.78	(−1.49, 0.07)	0.032	0.67	(−1.56, 0.22)	0.14	0.13	(−0.69, 0.95)	0.75
MAS distal score	81	−0.11	−0.46	(−1.38, 0.46)	0.032	- *	-	-	0.05	(−1.03, 1.13)	
MAS proximal score	83	−0.31	−2.16	(−3.60, −0.71)	0.004	−1.85	(−3.78, 0.072)	0.06	−0.25	(−1.94, 1.445)	0.78
Duration											
Time since onset	84	0.10	0.083	(−0.10, 0.27)	0.37	0.11	(−0.26, 0.49)	0.48	0.065	(−0.40, 0.53)	0.78
**TREATMENT CHARACTERISTICS**											
No muscles injected											
Muscle count	85	−0.032	−0.13	(−1.07, 0.80)	0.77	−0.22	(−1.05, 1.48)	0.74	−0.17	(−1.40, 1.06)	0.79
BoNT-A dose											
Proportion max dose	85	−0.054	−0.022	(−0.11, 0.07)	0.62	-	-		0.025	(−0.094, 0.14)	0.68

* Model could not be fitted; CIs = confidence intervals; MAS = Modified Ashworth Scale; BoNT-A = botulinum toxin A.
